# Chronic treatment with cisplatin induces replication-dependent sister chromatid recombination to confer cisplatin-resistant phenotype in nasopharyngeal carcinoma

**DOI:** 10.18632/oncotarget.2210

**Published:** 2014-07-12

**Authors:** Wen-Pin Su, Sen-Huei Hsu, Cheng-Kuei Wu, Song-Bin Chang, Yi-Ju Lin, Wen-Bin Yang, Jan-Jong Hung, Wen-Tai Chiu, Shun-Fen Tzeng, Yau-Lin Tseng, Jang-Yang Chang, Wu-Chou Su, Hungjiun Liaw

**Affiliations:** ^1^ Department of Internal Medicine, National Cheng Kung University Hospital, College of Medicine, National Cheng Kung University; ^2^ Graduate Institutes of Clinical Medicine, College of Medicine, National Cheng Kung University; ^3^ Department of Life Sciences, National Cheng Kung University, No.1 University Road, Tainan City 701, Taiwan; ^4^ Institute of Bioinformatics and Biosignal Transduction, National Cheng Kung University; ^5^ Department of Biomedical Engineering, National Cheng Kung University; ^6^ Department of Surgery, National Cheng Kung University Hospital, College of Medicine, National Cheng Kung University; ^7^ National Institute of Cancer Research, National Health Research Institutes, Taiwan; ^8^ Cancer Center, National Cheng Kung University Hospital, College of Medicine, National Cheng Kung University

**Keywords:** cisplatin, cisplatin resistant phenotype, homologous recombination, template-switching, Fanconi anemia, nasopharyngeal carcinoma

## Abstract

Cisplatin can cause intrastrand and interstrand crosslinks between purine bases and is a chemotherapeutic drug widely used to treat cancer. However, the major barrier to the efficacy of the treatment is drug resistance. Homologous recombination (HR) plays a central role in restoring stalled forks caused by DNA lesions. Here, we report that chronic treatment with cisplatin induces HR to confer cisplatin resistance in nasopharyngeal carcinoma (NPC) cells. A high frequency of sister chromatid exchanges (SCE) occurs in the cisplatin-resistant NPC cells. In addition, several genes in the Fanconi anemia (FA) and template switching (TS) pathways show elevated expression. Significantly, depletion of HR gene BRCA1, TS gene UBC13, or FA gene FANCD2 suppresses SCE and causes cells to accumulate in the S phase, concomitantly with high γH2AX foci formation in the presence of low-dose cisplatin. Consistent with this result, depletion of several genes in the HR, TS, or FA pathway sensitizes the cisplatin-resistant NPC cells to cisplatin. Our results suggest that the enhanced HR, in coordination with the FA and TS pathways, underlies the cisplatin resistance. Targeting the HR, TS, or FA pathways could be a potential therapeutic strategy for treating cisplatin-resistant cancer.

## INTRODUCTION

Cisplatin and related compounds are chemotherapeutic drugs widely used to treat lung, ovarian, head and neck, bladder, and testicular cancer [1]. These agents have proven to be quite effective in treating cancer; however, the major barrier to the efficacy of these treatments is drug resistance. Multiple pathways that mediate intrinsic or acquired resistance to drugs have been identified, including increased efflux pumps, increased detoxification, and decreased import [2]. Recently, a few studies have started to show that enhanced DNA damage repair may also underlie the mechanism of drug-resistance [3-6]. However, very few DNA repair genes have been validated *in vivo* so far. Cisplatin binds to DNA to form intrastrand and interstrand crosslinks between purine bases. When DNA replication machinery encounters the cisplatin-caused DNA damage, it stalls DNA replication, and prolonged stalling of replication forks eventually leads to fork collapse and the generation of replication-dependent DNA double-strand breaks (DSBs) [7, 8]. To prevent stalling of replication caused by cisplatin, cells have evolved the Fanconi anemia (FA) pathway in coordination with post-replication repair (PRR) and homologous recombination (HR) to resolve the cisplatin-caused DNA damage [9, 10].

The FA pathway is essential to the repair of interstrand cross-links caused by cisplatin and related compounds [9, 10]. This pathway is composed of at least 15 genes, which are named FANCA through FANCP. FANCA/B/C/E/F/G/L/M form the core ubiquitin E3 ligase, which promotes the monoubiquitination of FANCD2 and FANCI in response to crosslink-types of DNA damage during the S phase [11]. Monoubiquitination of FANCD2 and FANCI is the key regulatory step in the pathway, which recruits several nucleases including FAN1, SLX4, MUS81-EME1, and XPF-ERCC1 to the site of repair to initiate the incision [12-16]. This process generates DSBs which are subsequently repaired by HR. Importantly, several HR components are part of the FA pathway. BRCA2, which is also called FANCD1, is one of FA subunit. FANCN, also called PALB2, binds and regulates localization of BRCA2. These proteins facilitate the loading of RAD51 to initiate the HR process [8-10, 17, 18].

Post-replication repair (PRR) is known to prevent prolonged stalling of replication forks [19, 20]. The unique feature of PRR is that it bypasses DNA lesions to resolve arrested forks without removing the actual damage [19]. Recent studies in yeast and higher eukaryotes have revealed that the regulation of PRR occurs via the sumolation or ubiquitination of PCNA [21-30]. PRR can be divided into two sub-pathways, the translesion synthesis (TLS) and template switching (TS) pathways. The importance of the TLS pathway has been extensively studied. TLS utilizes low fidelity DNA polymerases (TLS polymerases) such as Polη, Polι, Polζ, Polκ, and Rev1, and allows cells to replicate over DNA lesions [31]. Indeed, recent studies have demonstrated that these TLS polymerases are involved in bypassing the unhooked cross-linked nucleotides, and thus restore the nascent strand [32, 33]. In addition, deletion of TLS polymerases sensitizes cells to cisplatin [31, 34, 35]. Suppression of Rev3, the catalytic subunit of Polζ, sensitizes drug resistant lung tumors to chemotherapy [4]. Importantly, TLS polymerase polζ is responsible for the variant form of xeroderma pigmentosum (XPV), an inherited disorder associated with high incidence of sunlight-induced skin cancers [36].

By contrast, the mechanism of the TS pathway is poorly understood in mammalian cells. It remains elusive whether the TS pathway is incorporated into the FA pathway, similar to the TLS pathway. The TS pathway is mediated by the K63-linked polyubiquitin chain at K164 of PCNA. The K63-linked polyubiquitin is catalyzed by the E2 ubiquitin-conjugating enzymes UBC13 and MMS2, and E3 ubiquitin ligases SHPRH and HLTF [25, 27, 28, 37, 38]. The K63-linked polyubiquitin chain is thought to function in signal transduction pathways presumably by recruiting proteins involved in the TS mechanism. Although the mechanism of the TS pathway is not clear, one model suggests that a stalled fork caused by the DNA lesions might undergo the fork regression that allows the original template strands to anneal, thereby extruding newly synthesized DNA strands as a short duplex, a structure similar to a “chicken foot” [38]. However, more and more recent evidence supports an alternative template switching model [17, 33]. The nascent strand impeded by DNA lesions could invade the opposite undamaged sister chromatid and replicate DNA by using the undamaged sister strand as a template, thus forming a double Holliday junction. The resolution of the Holliday junction leads to the generation of sister chromatid exchanges (SCE) [17, 33, 39]. Indeed, recent discoveries demonstrate that HLTF is involved in the strand invasion mechanism that can promote gap filling during replication of damaged DNA [40]. In addition, several lines of evidence have shown that HR components, such as RAD51, MRE11, BRCA1, and BRCA2, are involved in the resolving of stalled forks [33, 41-44].

Therefore, both the FA and TS pathways converge to the HR components to resolve stalled forks caused by DNA damage. However, it remains elusive whether the TS pathway is involved in restoring stalled forks caused by cisplatin and whether the TS pathway is enhanced in the drug-resistant phenotype of cancer. Here, we report that chronic treatment of cisplatin can induce elevated expression of several genes in the HR, TS, and FA pathways in nasopharyngeal carcinoma (NPC) cells. UBC13, HLTF, and SHPRH in the TS pathway, RAD51 and BRCA1 in the HR pathway, and FANCD2 in the FA pathway are highly expressed in cisplatin-resistant nasopharyngeal carcinoma (NPC) cells. Consistent with these results, these cisplatin resistant cells exhibit the elevated SCE frequency. Depletion of the HR gene BRCA1, the TS gene UBC13, or the FA gene FANCD2, dramatically reduces SCE frequency. In addition, these HR-, TS- or FA-deficient cells are sensitive to cisplatin and arrest in the S phase in chronic low-dose treatment with cisplatin. Since chronic low-dose treatment with cisplatin induces DNA damage and cell apoptosis in these HR-, TS- or FA-deficient cells, our results suggest the importance of HR in coordination with the TS and FA pathways in cell survival, as well as the importance of damage bypass mechanism during chronic low-dose treatment with cisplatin. Our results further suggest that targeting the HR, TS or FA pathways could be a potential therapeutic strategy for treating cisplatin-resistant cancer.

## RESULTS

### Cisplatin-resistant HONE6 cells are resistant to various DNA damaging agents

**Figure 1:**
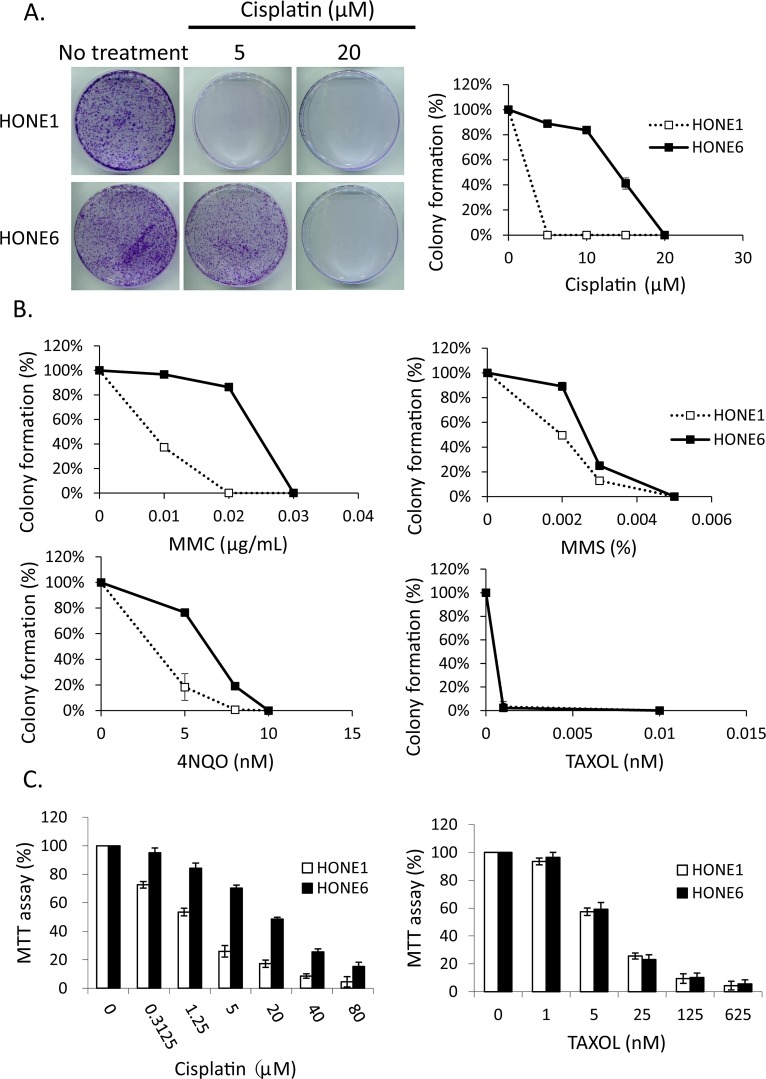
The cisplatin resistant NPC cells, HONE6 cells, are resistant to DNA damage agents (A) The colony formation assay. HONE1 and HONE6 cells were chronically treated with cisplatin as indicated and incubated for 10 days. The resulting colonies were stained with 1% crystal violet. Relative viability was determined by colonies arising from chronically treated cells relative to no treatment control cells. Each value represents the mean ± standard deviation from at least three experiments. (B) HONE1 and HONE6 cells were chronically treated with chemicals as indicated and incubated for 10 days. Relative viability was determined as mentioned above. (C) Cytotoxicity assay of HONE1 and HONE6 cells. Cells were treated with various concentrations of cisplatin or paclitaxel for 72 hours. Cytotoxicity was determined by the MTT assay.

To determine whether enhanced DNA damage repair may underlie the mechanism of cisplatin-resistance, we chose to use the cisplatin-resistant NPC cell line, HONE6, derived from a previously described NPC cell line, HONE1 [45]. HONE6 cells were generated by chronic treatment of HONE1 cells with low-dose cisplatin [46]. As shown in Figure 1A, HONE6 cells are resistant to chronic treatment of 5, 10, and 15 μM cisplatin, whereas HONE1 cells are sensitive to chronic treatment of cisplatin, with 5μM cisplatin being sufficient to kill all HONE1 cells.

To further explore the cisplatin-resistant phenotype that is not specific to cisplatin, but a phenomenon specific to DNA damage, we treated the cisplatin-resistant HONE6 cells with various DNA damaging agents, including mitomycin C (MMC), methylmethane sulfonate (MMS), and 4-nitroquinoline-1-oxide (4NQO). The FA pathway is involved in repairing the MMC-caused DNA damage, whereas the TS pathway is involved in repairing MMS- and 4NQO-caused DNA damage. Cell survival was determined by the colony formation assay. HONE6 cells are also resistant to these DNA damaging agents (Figure 1B), suggesting that the drug-resistant phenotype is specific to DNA damage. In support of this notion, we also found that HONE1 and HONE6 cells are equally sensitive to paclitaxel (Taxol), a mitotic inhibitor used in chemotherapy (Figure 1B). This result is also confirmed by the cytotoxicity assay [3-(4,5-dimethylthiazol-2yl)-2,5-diphenyltetrazolium bromide (MTT) assay] (Figure 1C). Paclitaxel is known to interfere with microtubule breakdown during mitosis, thus arresting cells in the G2/M phase and resulting in cell apoptosis. Therefore, our results indicate that HONE6 cells may acquire an enhanced DNA repair system related to the S phase progression.

### Cisplatin induces DNA damage both in cisplatin sensitive HONE1 and resistant HONE6 cells

**Figure 2:**
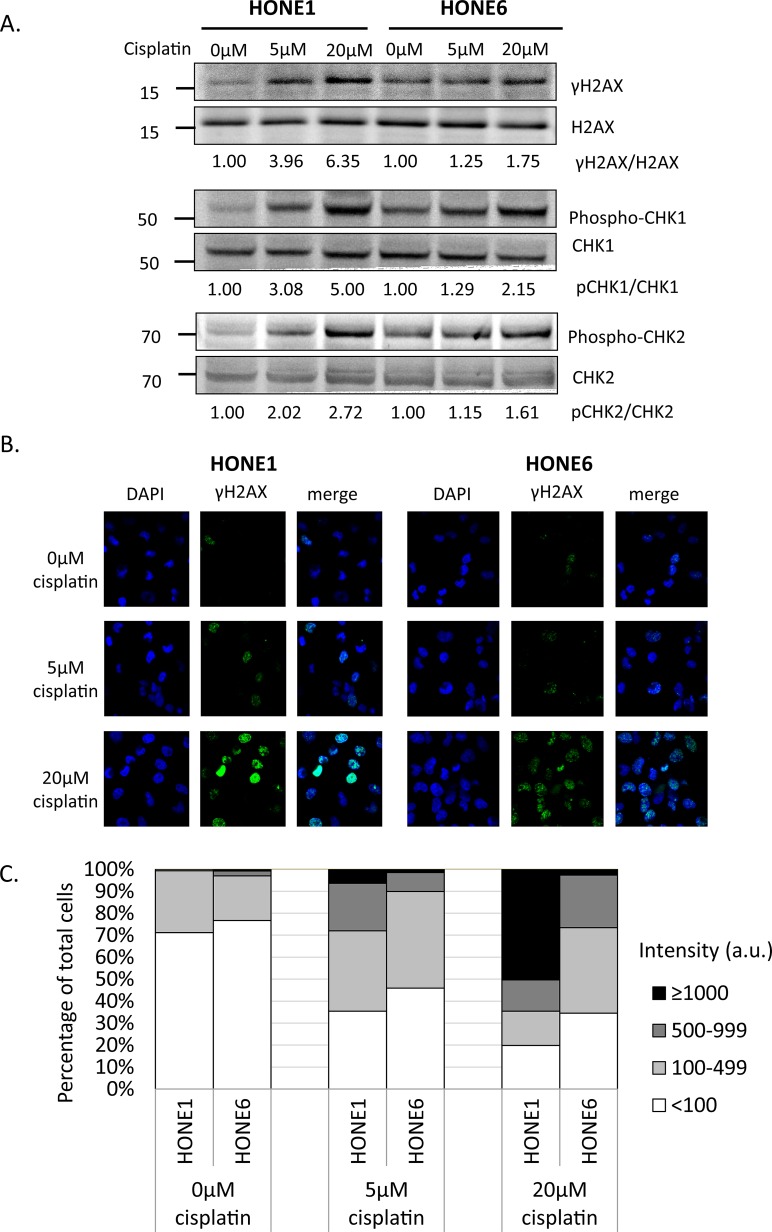

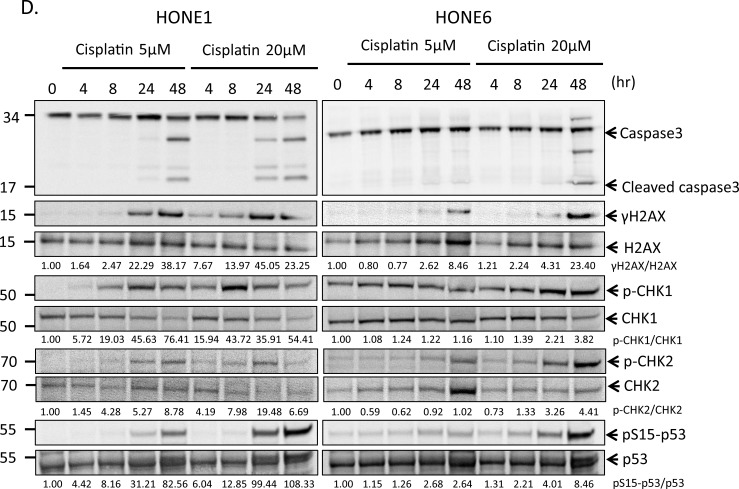
Cisplatin induces DNA damage both in HONE1 and HONE6 cells (A) HONE1 and HONE6 cells were chronically treated with 5μM or 20μM of cisplatin for four hours. Cells were harvested and subjected to Western blotting with antibodies as indicated. The γH2AX/H2AX, p-CHK1/CHK1, and p-CHK2/CHK2 ratios were indicated. (B) HONE1 and HONE6 cells were fixed and immunostained with γH2AX antibody. (C) The intensity of γH2AX in each cell was quantified from image (B) using an OLYMPUS FLUOVIEW: FV10-ASW software. More than 100 cells of each cell line were quantified. (D) Kinetics of DNA damage response between HONE1 and HONE6 cells. HONE1 and HONE6 were treated with 5μM or 20μM of cisplatin for 48 hours. Cells were harvested at the indicated time and subjected to Western blotting with specific antibodies as indicated.

To determine whether cisplatin treatment induces DNA damage both in cisplatin-sensitive HONE1 and cisplatin-resistant HONE6 cells, we examined the level of γH2AX by Western blotting and intensity of γH2AX foci in cells. HONE1 and HONE6 cells were treated with mock, 5μM, or 20μM cisplatin for four hours, followed by Western blot and fluorescence microscopy analysis. 5μM cisplatin indeed causes DNA damage in HONE1, but such treatment causes DNA damage to a lesser extent in HONE6 cells, as judged by γH2AX induction in Western blot and the intensity of γH2AX foci in cells (Figure 2A, B, and C). 20μM cisplatin treatment further increases γH2AX and the intensity of γH2AX, with HONE1 cells showing much stronger intensity of γH2AX than HONE6 cells (Figure 2A, B, and C). To determine whether DNA damage response plays a role in the cisplatin-resistant phenotype of HONE6 cells, we monitored the kinetics of γH2AX and the activation of CHK1, CHK2, and TP53 (p53) in response to cisplatin over time between HONE1 and HONE6 cells (Figure 2D). We found that the basal level of γH2AX, phospho-CHK1 (S345), phospho-CHK2 (T68), and phospho-TP53 (S15) are higher in HONE6 than in HONE1 cells (Figure 2A and D). However, 5μM cisplatin induces much stronger intensity of γH2AX, phospho-CHK1 (S345), phospho-CHK2 (T68), and phospho-TP53 (S15) in HONE1 cells than in HONE6 cells. Importantly, the phospho-TP53 (S15) correlates with apoptosis as judged by the cleavage form of caspase3 (Figure 2D). The intensity of γH2AX, phospho-CHK1 (S345), phospho-CHK2 (T68), and phospho-TP53 (S15) is in a time- and dose-dependent manner both in HONE1 and HONE6 cells (Figure 2D). Our results indicate that HONE6 acquires enhanced repair system to resolve stalled forks caused by cisplatin. By contrast, HONE1 cells encounter high frequency of stalled forks and collapse of forks caused by cisplatin, thus resulting in dramatically high activation of CHK1, CHK2, and TP53 and cell apoptosis. However, high dose of cisplatin (20μM) could also overwhelm the repair system in HONE6, causing collapse of forks and cell apoptosis.

### The cisplatin-resistant HONE6 cells can progress through the S phase in low-dose cisplatin treatment

**Figure 3:**
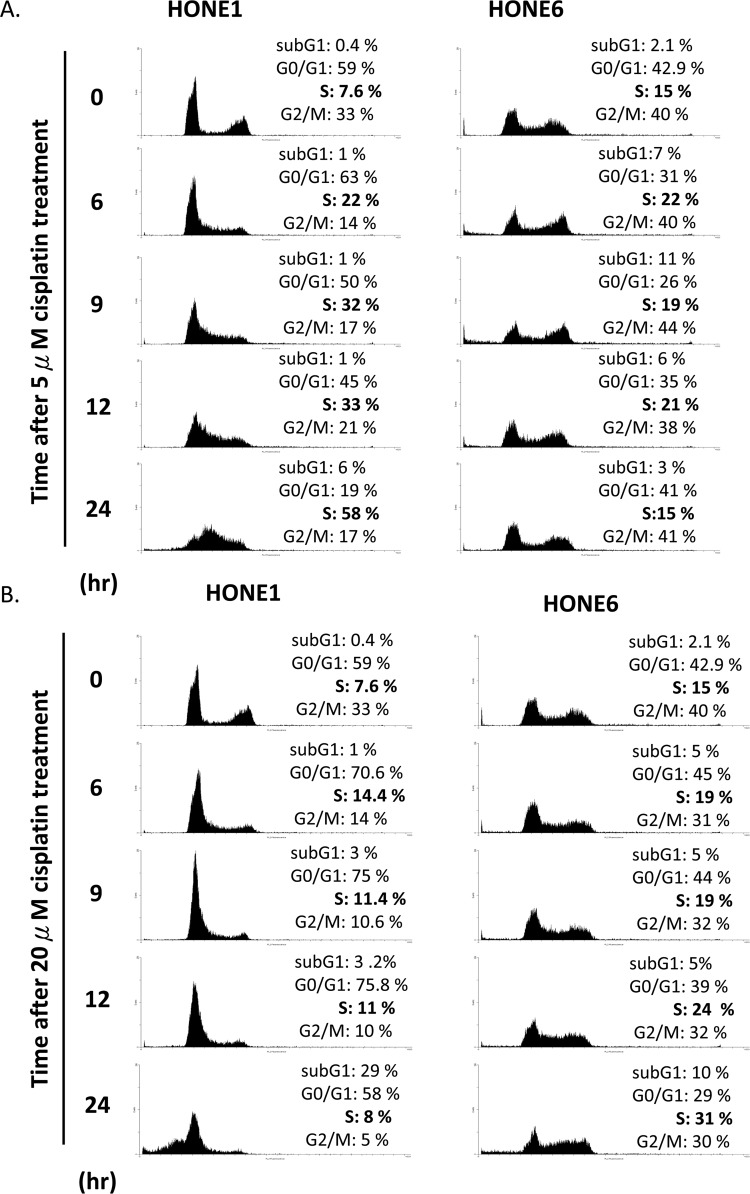
Cisplatin resistant HONE6 cells can progress through the S phase in the low-dose (5μM) cisplatin treatment (A) Flow cytometry of HONE1 and HONE6 cells in the presence of low-dose (5μM) cisplatin. Cells were chronically treated with cisplatin for 24 hours. (B) HONE1 and HONE6 cells were chronically treated with 20μM cisplatin for 24 hours. The cell cycle progression was determined by flow cytometry.

We further examined the cell cycle progression in HONE1 and HONE6 cells in the presence of 5μM and 20μM cisplatin treatments (Figure 3). HONE1 cells accumulate in the S phase in the presence of 5μM cisplatin, with 58% of cells arrested in the S phase at 24 hours after treatment (Figure 3A). 20μM cisplatin strongly arrests HONE1 cells in the G1 phase and 29% of HONE1 cells undergo apoptosis at 24 hours after treatment, as judged by cells in the sub-G1 phase (Figure 3B). By contrast, HONE6 cells can progress through the cell cycle without arresting in the S phase in the presence of 5μM cisplatin (Figure 3A). However, 20μM cisplatin treatment arrests HONE6 cells in the S phase, with 31% of cells accumulating in the S phase at 24 hours after treatment (Figure 3B). Since FA and TS are two major pathways to resolve stalled forks caused by DNA damage, and since HR is essential for both of these pathways, it appears that HONE6 cells may acquire enhanced HR, TS and FA pathways. However, 20μM cisplatin treatment could result in severe DNA lesions that overwhelm the HR, TS, and FA pathways, resulting in the accumulation of HONE6 cells in the S phase.

### Genes in the HR, TS, and FA pathways are highly expressed in HONE6 cells

**Figure 4:**
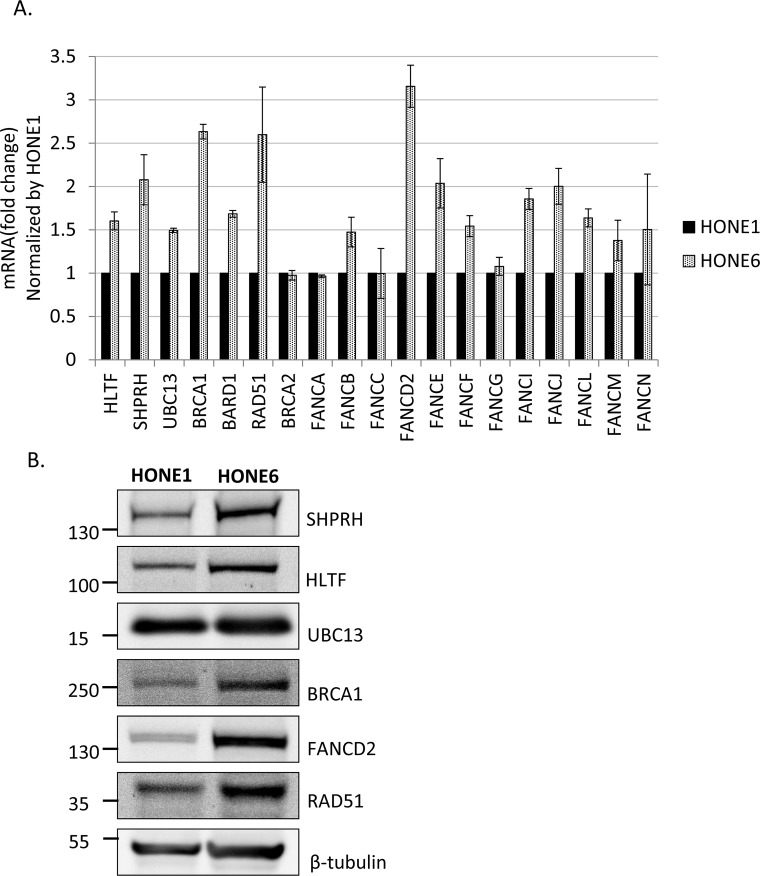
Several genes in the FA, HR, and TS pathway are overexpressed in cisplatin resistant HONE6 cells (A) The expression level of each gene in HONE1 and HONE6 cells was determined by qRT-PCR and normalized by the level in HONE1 cells. The expression of β-actin (ACTB) was used as an internal control. The expression level of each gene in HONE1 or HONE6 was normalized by the level of ACTB in each cell. (B) The Western blot was immunostained with specific antibodies as indicated.

To determine whether HR is induced in HONE6 cells, we examined the expression levels of HR genes in HONE6 cells by the quantitative reverse transcription polymerase chain reaction (qRT-PCR) and Western blotting. Indeed, genes in the HR pathway, including BRCA1, BARD1, and RAD51, are highly expressed in HONE6 cells, as compared with in HONE1 cells, as judged either by qRT-PCR or Western blotting (Figure 4).

Given that both the FA and TS pathways utilize HR components to resolve stalled forks, we also checked the expression of several genes in the FA and TS pathways. The TS genes, including SHPRH, HLTF, and UBC13, and the FA gene, including FANCB, FANCD2, FANCE, FANCF, FANCI, FANCJ, FANCL, FANCM, and FANCN have higher expression level in HONE6 cells than in HONE1 cells (Figure 4A). Previous studies have shown that monoubiquitination of FANCD2 and FANCI is the key regulatory step in the FA pathway, and FANCL and FANCI are sufficient for monoubiquitination of FANCD2 *in vitro* [18, 47]. Our results show that these key regulators, FANCD2, FANCI, and FANCL, are highly expressed in HONE6 cells, suggesting these key regulators of the FA pathway could play an important role in the cisplatin-resistant phenotype of HONE6 cells.

Therefore, Consistent with the results of flow cytometry and the phenotype of resistance to broad types of DNA damaging agents, HONE6 cells may acquire the enhanced HR in coordination with TS and FA to resolve stalled forks caused by DNA damage.

### Cisplatin-resistant HONE6 cells show elevated SCE frequency

**Figure 5:**
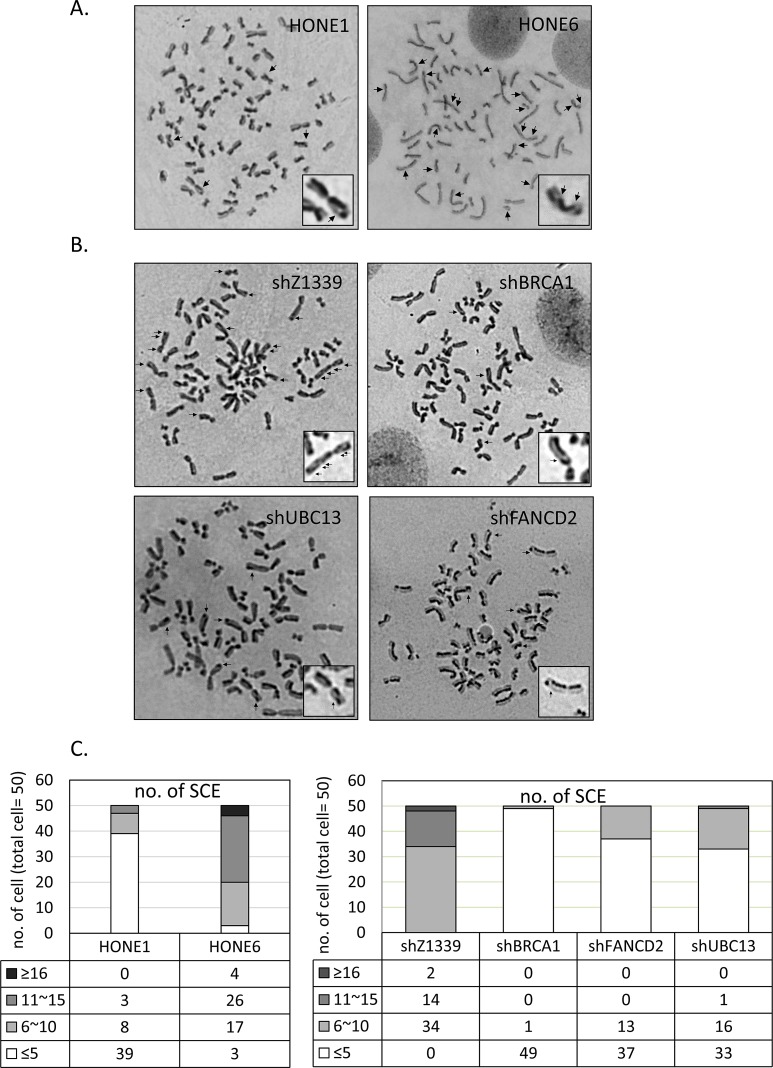
Cisplatin resistant HONE6 cells show elevated frequencies of sister chromatid exchange (SCE) (A) The SCE analysis of HONE1 and HONE6 cells. SCE are indicated by arrows. SCE was scored in 50 metaphases of HONE1 and HONE6 cells as indicated in (C). (B) The SCE analysis of control (shZ1339), BRCA1-(shBRCA1), FANCD2- (shFANCD2), and UBC13- (shUBC13) deficient cells. SCE was scored in 50 metaphases of each cell lines as indicated in (C).

To determine whether the enhanced HR in coordination with TS and FA pathways are responsible for the cisplatin-resistant phenotype, we performed a sister chromatid exchange (SCE) analysis to determine the HR efficiency (Figure 5). Strikingly, HONE6 cells show elevated SCE frequency. More than 90% of HONE6 cells showed more than 10 SCE, whereas 78% of HONE1 cells showed less than 5 SCE (Figure 5A and C). These results suggest that cisplatin-resistant HONE6 cells indeed acquired the enhanced HR pathway.

**Figure 6:**
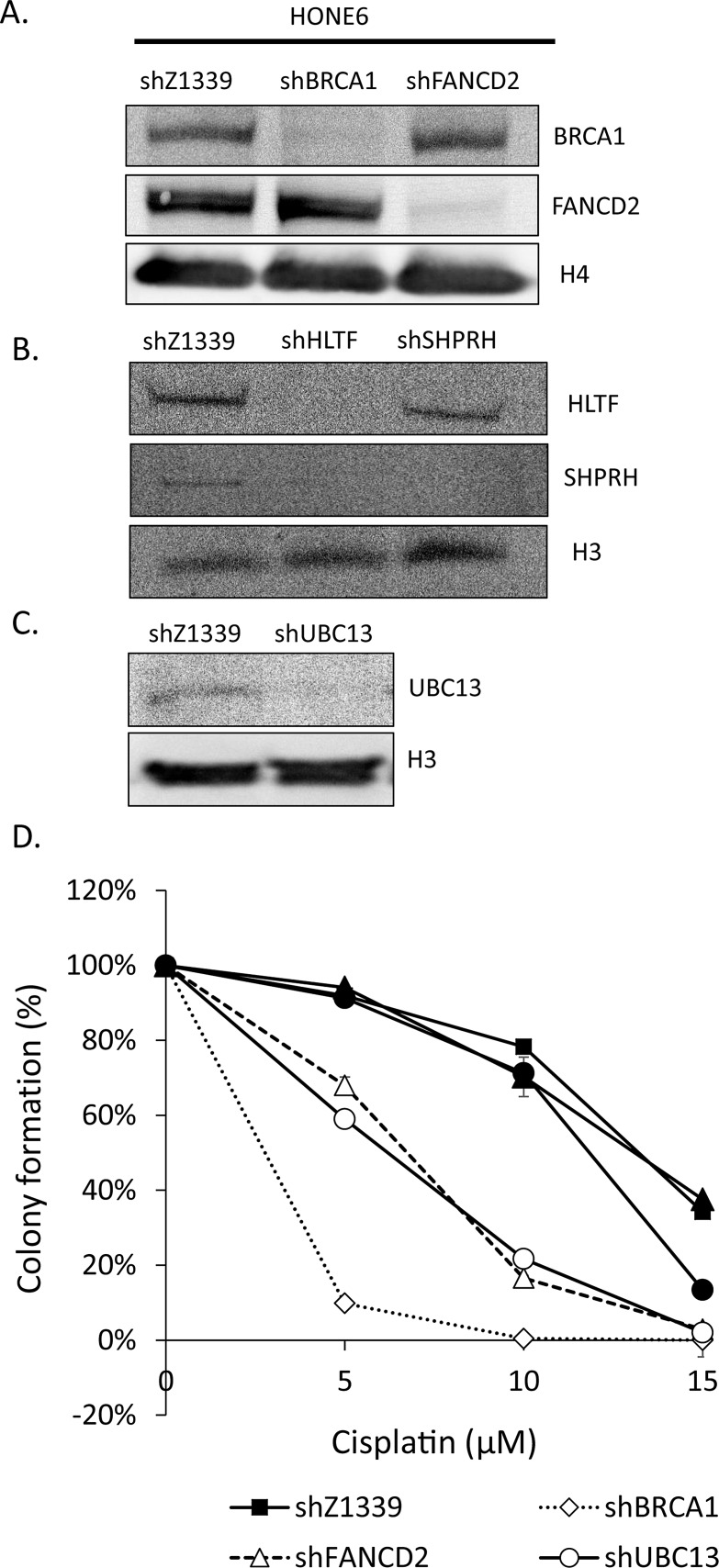
TS- or HR-deficient HONE6 cells are sensitive to cisplatin (A)(B)(C) HLTF, SHPRH, UBC13, BRCA1, and FANCD2 are depleted by the shRNA packed lentivirus in HONE6 cells. The scramble shRNA, shLacZ1339 was used as a control. Cell lysates were subjected to Western blotting with specific antibodies as indicated. (D) Cell viability was determined by the colony formation assay.

To further verify our hypothesis, we depleted the expression of the HR gene BRCA1, the TS gene UBC13, or the FA gene FANCD2, by shRNA packed lentivirus (Figure 6A and C). The scramble shRNA, shZ1339, was used as the control. As shown in Figure 5B and C, SCE frequency was dramatically reduced in the BRCA1-, UBC13-, or FANCD2-depleted cells, suggesting that the HR, TS, and FA pathways collaborate to contribute to cisplatin resistant-phenotype in HONE6 cells.

### Depletion of HR, TS, or FA genes sensitizes HONE6 cells to cisplatin

Given that the enhanced HR in coordination with the TS and HR pathways underlies the cisplatin-resistant phenotype, we reasoned that disruption of the HR, TS, or FA pathway would sensitize cells to the cisplatin treatment. Therefore, we depleted the expression of TS genes, including HLTF, SHPRH, and UBC13, the HR gene BRCA1, and the FA gene FANCD2, using the shRNA packed lentivirus, as mentioned previously (Figure 6A, B, and C). These shRNA can efficiently deplete the expression of HLTF, SHPRH, UBC13, BRCA1, and FANCD2 (Figure 6A, B, and C). Interestingly, depletion of HLTF also results in depletion of SHPRH (Figure 6B). The control cells have 95% cell survival in 5μM cisplatin and 90% cell survival in 10μM cisplatin (Figure 6D). By contrast, the UBC13-, BRCA1-, or FANCD2-deficient cells are dramatically sensitive to cisplatin, with 59%, 10%, and 68% cell survival rates in 5μM cisplatin, and 22%, 0%, and 17% cell survival rates in 10μM cisplatin, respectively. Therefore, depletion of UBC13-, BRCA1-, or FANCD2 greatly sensitizes HONE6 cells to cisplatin, but the level of sensitivity does not reach to that of HONE1 cells, since 5μM cisplatin kills all HONE1 cells.

Depletion of SHPRH has no significant effect on the viability of HONE6 cells in cisplatin relative to control cells. This could be due to the functional redundancy of SHPRH and HLTF. However, depletion of HLTF shows decreasing viability in chronic treatment with 15 μM of cisplatin (Figure 6D). By checking the expression of SHPRH and HLTF in the HLTF-depleted cells, we found that depletion of HLTF can simultaneously deplete the expression of SHPRH (Figure 6B), resulting in more sensitive to cisplatin than cells with silencing of SHPRH only (Figure 6D). Our results thus provide evidence that SHPRH and HLTF are functional redundant proteins.

### Depletion of the HR, TS, or FA pathways accumulates cells in the S phase and results in elevated DSBs after chronic treatment with low-dose (5μM or 10μM) cisplatin

**Figure 7:**
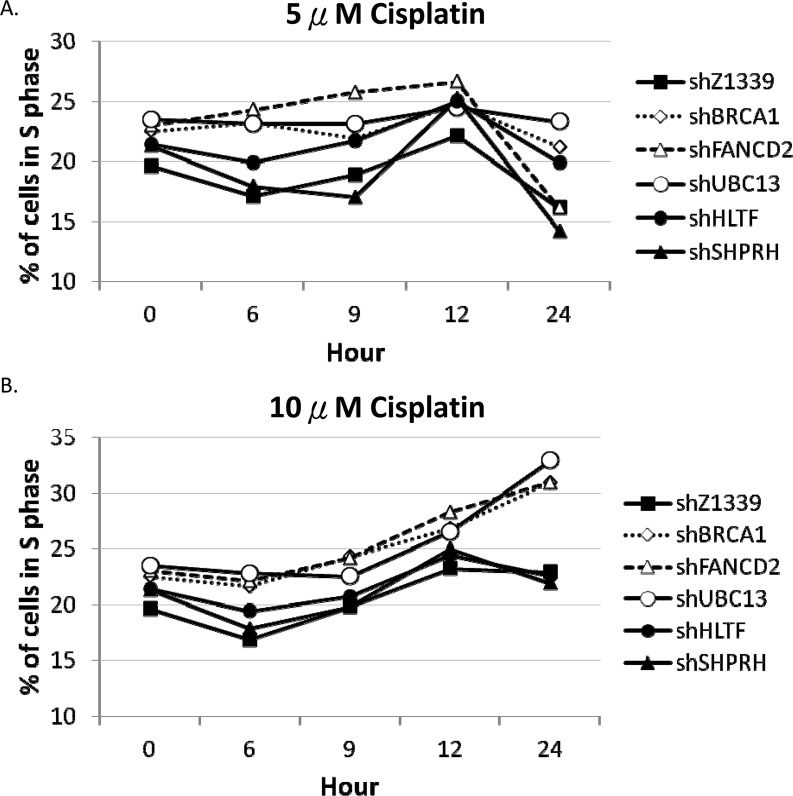
BRCA1-, FANCD2-, and UBC13-deficient cells accumulate in the S phase in the presence of 10μM cisplatin treatment Fractions of the S phase cells were determined by flow cytometry of HONE1 and HONE6 cells in the presence of 5μM (A) or 10μM (B) of cisplatin. Cells were chronically treated with cisplatin for 24 hours.

To further determine whether HR, TS and FA genes collaborate to resolve stalled forks, we monitored the cell cycle progression of the HR-, TS- or FA-deficient cells by flow cytometry. The HLTF-, SHPRH-, UBC13-, BRCA1-, or FANCD2-depleted cells were chronically treated with 5μM or 10μM cisplatin, and the cell cycle progression was monitored at 6, 9, 12, and 24 hours during the chronic treatment. As shown in Figure 7, the control cells can progress through the cell cycle during chronic treatment with cisplatin. However, the BRCA1-, FANCD2-, or UBC13-deficient cells start to accumulate in the S phase during chronic treatment with low-dose (5μM or 10μM) cisplatin (Figure 7A), while the arrest in the S phase is significantly increased in 10μM cisplatin treatment (Figure 7B). Therefore, the BRCA1-, FANCD2-, or UBC13-deficient cells encounter obstacles to progressing through the S phase during chronic treatment with low-dose cisplatin, resulting in cell arrest in the S phase.

**Figure 8:**
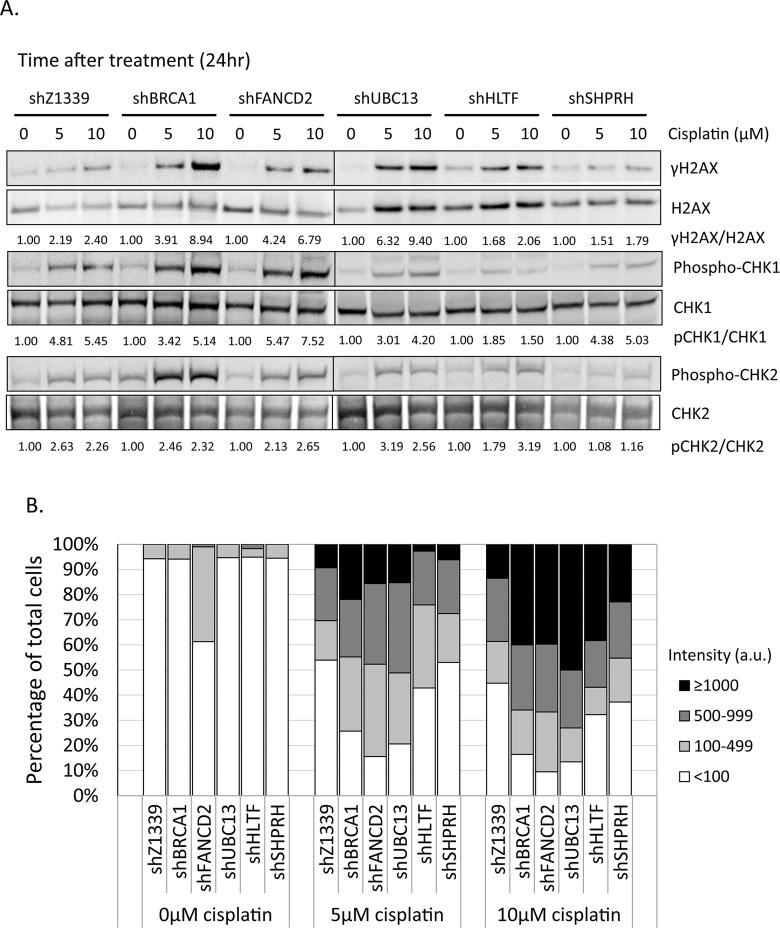

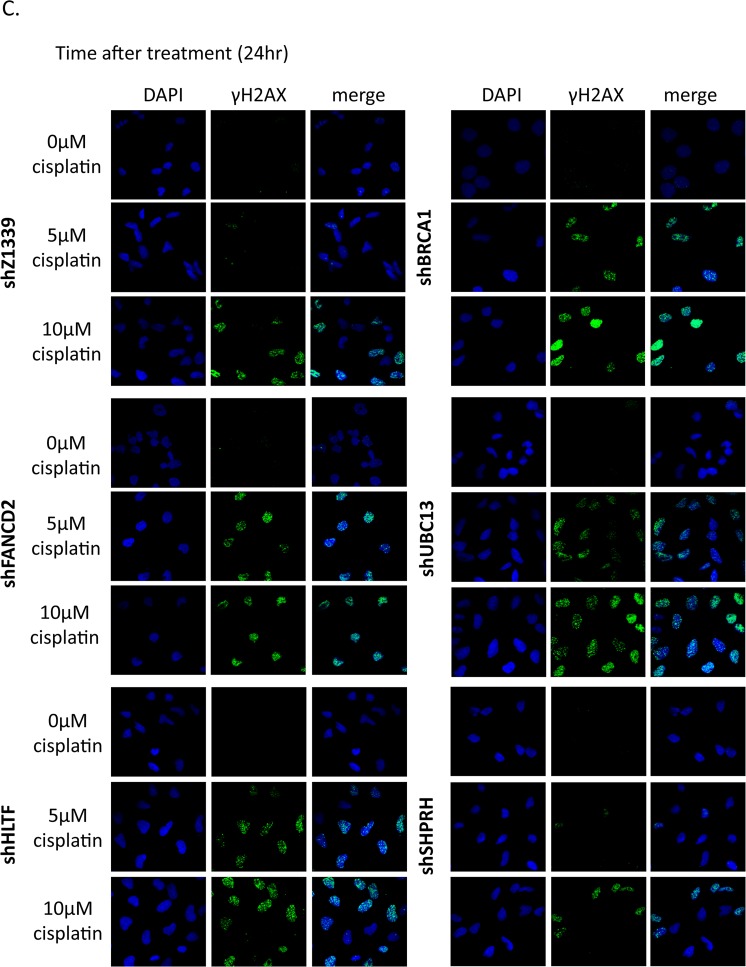
γH2AX is increased in the FA-, HR-, and TS-deficient cells in the presence of cisplatin treatment (A) The gene-depleted HONE6 cells were treated with 5μM or 10μM of cisplatin for 24 hours. Cell lysates were subjected to Western blotting with specific antibodies as indicated. The γH2AX/H2AX, pCHK1/CHK1, and pCHK2/CHK2 ratio was indicated. (B) The intensity of γH2AX in each cell was quantified from image (C) using an OLYMPUS FLUOVIEW: FV10-ASW software. More than 100 cells of each cell line were quantified. (C) The γH2AX foci formation of the control (shZ1339), UBC13- (shUBC13), HLTF- (shHLTF), SHPRH- (shSHPRH), BRCA1- (shBRCA1), and FANCD2- (shFANCD2) deficient cells was determined by the fluorescence microscopy with specific anti- γH2AX antibody.

To determine whether more DSBs are generated in cells due to the collapse of replication forks, we monitored the induction of γH2AX in cells during chronic treatment with low-dose cisplatin. Depletion of UBC13, BRCA1, or FANCD2 dramatically increased the intensity of γH2AX in terms of expression level and number of cells with γH2AX foci during treatment with low-dose cisplatin (Figure 8A, B, and C). Greater than 70% of these UBC13-, BRCA1- or FANCD2-deficient cells exhibited strong γH2AX intensity (>100 a.u.) in 5μM cisplatin and the level of intensity was further enhanced in 10μM cisplatin treatment, with more than 40% of cells showing greater than 1000 a.u. fluorescent intensity of γH2AX (Figure 8B). The phospho-CHK1 and phospho-CHK2 is also induced, suggesting that stalled forks and DSBs occur in these cells (Figure 8A).

Consistent with the survival curve, depletion of SHPRH or HLTF shows very similar γH2AX, phospho-CHK1, and phospho-CHK2 induction patterns to control cells. Functional redundancy of SHPRH and HLTF, or even the existence of additional E3 ligases might attribute to the minor phenotypes of the SHPRH- or HLTF-depleted cells.

## DISCUSSION

Drug resistance is the major obstacle for chemotherapy. Previous studies have identified several genes in the HR, nucleotide excision repair (NER), and TLS pathways that play an important role in the drug-resistant phenotype of cancer. In this study, we identified a new DNA repair pathway, the TS pathway, in the cisplatin-resistant phenotype of cancer. We demonstrated that chronic treatment with low-dose cisplatin induces the HR in coordination with the TS and FA pathways to confer the drug-resistant phenotype of cancer. Most importantly, the efficiency of resolving stalled forks caused by DNA damage is essential to the cisplatin resistant phenotype. In support of this notion, the cisplatin-resistant NPC cells, HONE6 cells, show elevated SCE frequency when compared to their cisplatin-sensitive precursors, HONE1 cells (Figure 5). Depletion of the HR gene BRCA1, the TS gene UBC13, or the FA gene FANCD2, suppresses SCE in HONE6 cells (Figure 5). In addition, these HR-, TS- or FA-deficient HONE6 cells are sensitive to cisplatin (Figure 6) and accumulate in the S phase in the presence of low-dose cisplatin (Figure 7), concomitantly with elevated γH2AX in cells (Figure 8). Our results suggest that HR, in coordination with TS and FA components, participates in fork progression, thus playing an important role in the cisplatin-resistant phenotype of cancer.

Previous studies have extensively addressed the importance of the FA pathway in resolving cisplatin-caused DNA damage and the cisplatin-resistant phenotype. However, the information regarding the function and significance of the TS pathway in the drug-resistant phenotype remains elusive. In particular, what is the outcome of chronic treatment with low-dose cisplatin? Here, we discovered that chronic treatment with low-dose cisplatin induces the TS pathway in addition to the HR and FA pathways. Interestingly, a recent discovery also demonstrated the importance of the TS pathway during chronic low-dose of UV irradiation (CLUV) in yeast *Saccharomyces cerevisiae* [48]. In addition to the TS gene, the HR proteins RAD51, RAD52, and RAD54 are critical for recovery from CLUV irradiation in yeast [48]. We provide several lines of evidence that the TS pathway is particularly important to survival during chronic treatment with low-dose cisplatin in human cells and that HR components are also involved in the TS pathway. First, chronic treatment with low-dose cisplatin induces the expression of TS and HR genes to confer cisplatin resistance. Second, consistent with high expression of TS and HR genes, these cisplatin-resistant HONE6 cells can progress through the S phase in chronic treatment with low-dose cisplatin and exhibit elevated SCE frequency. Third, depletion of TS or HR genes sensitizes HONE6 cells to cisplatin and accumulates cells in the S phase in the presence of low-dose cisplatin. Fourth, these TS- or HR-deficient cells exhibit high levels of DSBs, as judged by the elevated γH2AX in these cells. Indeed, the TS pathway involves the strand invasion of a nascent strand into the undamaged sister chromatid, a process that generates a double Holliday junction [17, 33, 39]. Recently, HLTF has been shown to be involved in the strand invasion mechanism that can promote gap filling during replication of damaged DNA [40]. Therefore, HR proteins would be involved in resolving the Holliday junction and result in SCE. In support of this notion, we also found that a high SCE frequency occurs in HONE6 cells. Importantly, depletion of UBC13 or BRCA1 dramatically reduces SCE in HONE6 cells. Recent studies also support this notion. Several HR proteins, such as RAD51, MRE11, BRCA1, and BRCA2, are involved in resolving stalled forks [41-44].

Interestingly, depletion of either SHPRH or HLTF did not result in severe phenotype as much as depletion of UBC13. Functional redundancy of SHPRH and HLTF, or even the existence of additional E3 ligases could account for the discrepancy. Given the large size of human genome, functional redundancy might have evolved to ensure the successful completion of DNA replication in the presence of low levels of DNA damage. For example, there is only one E3 ligase, Rad5, in the TS pathway in yeast, whereas at least two orthologs of Rad5, SHPRH and HLTF, are evolved in human cells [38]. A recent study even suggests that additional E3 ligases might exist in the TS pathway in mammalian cells [24]. In addition, previous studies have shown that the E3 ligases, RNF8 and RNF168, can also interact with UBC13. The interaction between RNF8, RNF168, and UBC13 generates K63-linked polyubiquitin chains at H2A or γH2AX, which in turn recruit BRCA1 to sites of DNA damage [49-52]. Therefore, silencing of UBC13 can affect more biological pathways than silencing of HLTF and SHPRH, which could account for the reason why silencing of UBC13 results in more severe phenotype than silencing of SHPRH and HLTF. As to whether RNF8 and RNF168 can also ubiquitinate PCNA, and whether RNF8 and RNF168 could be the missing E3 ligases in the TS pathway, further studies to generate the triple or quadruple knockout of SHPRH, HLTF, RNF8, and RNF168 might answer this question.

To determine whether DNA damage checkpoint plays a role in cisplatin-resistant phenotype of HONE6 cells, we monitored the kinetics of γH2AX, phospho-CHK1 (S345), phospho-CHK2 (T68), and phospho-TP53 (S15) in response to cisplatin over time in HONE1 and HONE6 cells (Figure 2D). Our results demonstrate that both HONE1 and HONE6 are not checkpoint-defective. Interestingly, we found that the basal level of γH2AX, phospho-CHK1 (S345), phospho-CHK2 (T68), and phospho-TP53 (S15) are higher in HONE6 than in HONE1 cells (Figure 2A and D). It could be due to the elevated SCE (because DSBs occur to initiate SCE). However, cisplatin-sensitive HONE1 cells respond to cisplatin more vigorously than cisplatin-resistant HONE6 cells, which shows much stronger intensity of γH2AX, phospho-CHK1 (S345), phospho-CHK2 (T68), and phospho-TP53 (S15) in HONE1 cells than in HONE6 cells. Our results support the notion that cisplatin-sensitive HONE1 cells encounter high frequency of stalled forks and collapse of forks caused by cisplatin, thus resulting in dramatically high activation of CHK1, CHK2, and TP53 and cell apoptosis. By contrast, HONE6 acquires enhanced repair system to resolve stalled forks caused by cisplatin, thus resulting in less activation of DNA damage response.

As to what is the suspected mechanism for the induction of these FA, TS, and HR genes in HONE6 cells? FOXM1 (forkhead box protein M1) is a transcription factor and has been shown to associate with cisplatin-resistance and DNA damage response in breast cancer [53-58]. We discovered that the expression level of FOXM1 is slightly increased in HONE6 cells compared to HONE1 cells, as judged by qRT-PCR and Western blotting (Figure S3). Therefore, FOXM1 could underlie the induction mechanism that induces FA, TS, and HR genes. As several other transcription factors, such as E2F, NF-kB, TIP60, Nrf2, GCF2, are also indicated in the cisplatin-resistant phenotype [59]. We cannot rule out the possibility that these transcription factors are also involved in the induction mechanism. Further investigation will be worthy of exploration to determine the mechanism.

In this study, we discovered that HR, in coordination with the TS and FA pathways, plays a central role in the cisplatin-resistant phenotype of cancer. Targeting these three pathways may thus provide a therapeutic strategy for treating cisplatin-resistant cancer.

## MATERIALS AND METHODS

### Cell culture

The nasopharyngeal carcinoma HONE1 cells were gifts from the distinguished investigator Jang-Yang Chang (NHRI, Taiwan) and were cultured in RPMI1640 supplemented with 5% FBS, 1% glutamine, and 1% penicillin/streptomycin [45, 46]. HONE6 cells were developed through chronic low-dose treatment of HONE1 using cisplatin containing medium (RPMI1640 supplemented with 5% FBS, 1% glutamine, 1% penicillin/streptomycin, and increasing dose to 5μM cisplatin) [46].

### Colony formation assay

Approximately 10^4^ cells were seeded in 100-mm dishes in duplicate. Subsequently, cells were chronically treated with cisplatin, MMC, MMS, and 4NQO, and incubated for 10 days. The resulting colonies were stained with 1% crystal violet (Sigma). Colonies were counted using a GeneTools software program (Syngene).

### Cytotoxicity assay

Cytotoxicity was determined by 3-(4,5-dimethylthiazol-2yl)-2,5-diphenyltetrazolium bromide (MTT) assay. 10^5^ cells were grown in culture plates for 24 hours and then treated with various concentrations of drugs for another 72 hours. 0.5 mg/ml MTT was added to each culture, followed by incubation at 37°C for 4 hours. Dimethyl sulfoxide (DMSO) was added to dissolve the converted dye of the released MTT. Absorbance was measured at a wavelength of 490 nm using a microplate reader. Cytotoxicity induced by each treatment was calculated as the percentage of viable cells by dividing the optical density of samples in drug-treated wells by that of the control wells.

### qRT-PCR analysis

Total RNA from HONE1, HONE6, or gene-depleted HONE6 cells was isolated using Trizol reagent (Invitrogen) and was subjected to reverse transcription using GoScript^TM^ reverse transcription system (Promega). The resulting cDNA samples were analyzed using the real-time PCR analysis (ABI StepOne Plus^TM^ Real-Time PCR Systems). The real-time PCR was performed in a 40μl reaction with 0.8μl of 10μM primers, 0.8μl of 50mM MgCl_2_, 20μl iQ^TM^SYBR green supermix (BIO-RAD), and 1μl cDNA. The primer sequences are following, BRCA1: 5′-AGC AGA ATG GTC AAC TGA TGA ATA-3′ and 5′-ACT GCT GCT TAT AGG TTC AGC TTT-3′; RAD51: 5′-CAG TGA TGT CCT GGA TAA TGT AGC-3′ and 5′-TTA CCA CTG CTA CAC CAA ACT CAT-3′; FANCD2: 5′-ATC TGC TAT GAT GAT GAA TGC TGT-3′; UBC13: 5′-CAA TGG CAG CCC CTA AAG TA-3′ and 5′-GTC TTC CAC TGC TCC GCT AC-3′; HLTF: 5′-GTG CAA TTT GCC TGG ATT CT-3′ and 5′-TAG CAT GTG GCT GCT CAT TC-3′; SHPRH: 5′-GCC AAA GCA CTC GTT TTC TC-3′ and 5′-TTG ATT TGG GGA TCA CGT TT-3′; ACTB: 5′-AGG CAT CCT CAC CCT GAA GTA-3′ and 5′-GGG ATA GCA CAG CCT GGA TAG-3′; FANCA: 5′- aaa ata taa tcc tga aag ggc aca-3′ and 5′- aat gat tag cat agg cct cag aac-3′; FANCB: 5′- tgg ttg ttg gag tga aaa cta cat-3′ and 5′- aca aag ctt tcc tct ttc ttg cta-3′; FANCC: 5′- tta gca tat gat gaa agc caa aaa-3′ and 5′- aga cct tga gtg aaa aga gca act-3′; FANCE: 5′- aga gtt act gtg ttg cct tgt gaa-3′ and 5′- ata ctt ggt cat cac tgt cag cat-3′; FANCF: 5′- gct tca atg gct ata gag aga acc-3′ and 5′- tat cac ctt cag gaa gtt gtt ctg-3′; FANCG: 5′- cta gag aga gtg ctg gag aca cag-3′ and 5′- gcc att cag ggt ctc tag taa caa-3′; FANCI: 5′- cag aaa gag tgt ttt gga agg aat-3′ and 5′- tta agt gtt tca cga gtt ctc tgc-3′; FANCJ: 5′- tct cca ctg gaa aag ata aac tcc-3′ and 5′- agt aat ctg agc aat ctg ctt gtg-3′; FANCL: 5′- act atg ctt cct gag tgc ttc ttt-3′ and 5′- gca taa caa att cca caa tcc ata-3′; FANCM: 5′- tat gct tat tgc cag gtt gta aga-3′ and 5′- cgg aac aat aag ctt ttc aac ttt-3′; FANCN: 5′- aaa aac ttt ata cct ggc act tcg-3′ and 5′- cca ctg cta cta act agc ctc ctc-3′; BRCA2: 5′- aaa caa caa tta cga acc aaa cct-3′ and 5′- cat cat ctg ctt gat cca ttt tag-3′; BARD1: 5′- aaa ttt gaa tgg gta aaa gca tgt-3′ and 5′- taa taa ggt tgt cct ttg gat ggt-3′; FOXM1: 5′- gat gtg aat ctt cct aga cca cct-3′ and 5′- aat tct cct ttt cct cca tct ctt-3′. The real-time PCR was started at 95°C for 3min, followed by 40 cycles at 95°C for 15sec, and 55°C for 45sec.

The expression of β-actin (ACTB) was used as an internal control. The expression level of each gene in HONE1 or HONE6 was normalized by the level of ACTB in each cell.

### shRNA lentiviral gene knockdown

HEK293T was used as packaging cells to generate shRNA encoding lentiviruses. Cells infected with lentiviruses were selected in 2μg/ml puromycin. All RNAi reagents were obtained from the National RNAi Core Facility, Academia Sinica. Sequences targeted by shRNAs were as follows: shZ1339: CGC GAT CGT AAT CAC CCG AGT (TRCN0000244984); shBRCA1: GAG TAT GCA AAC AGC TAT AAT (TRCN0000244984); shFANCD2: ATC ATG CAG CTG ATC AGT ATT (TRCN0000417689); shUBC13: AGA CAA GTT GGG AAG AAT ATG (TRCN0000368937); shHLTF: TGT GGT TGG ACT ACG CTA TTA (TRCN0000272562); shSHPRH: ACG GAA CCA GAA GCG CTA TAT (TRCN0000235922). Depletion of genes was verified by qRT-PCR and Western blotting.

### Flow cytometry

Cells were washed with PBS and fixed in ice-cold 70% ethanol overnight. Fixed cells were then treated with RNase at 37°C for 30 min. After incubation with propidium iodide solution (Invitrogen), cells were analyzed by flow cytometry (Cell Lab Quanta™ SC Flow Cytometer, Beckman Coulter).

### Western Blotting

10^6^ cells were washed and resuspended in lysis buffer [50mM Tris (pH7.5), 150 mM NaCl, 1mM EDTA, 0.1% Triton X-100, protease inhibitor cocktail (MD Biol)]. After sonication, cell lysates were added with Laemmli sample buffer and boiled for 5 minutes. Samples were separated on a 10% SDS-PAGE and transferred to a PVDF membrane. Protein blots were probed using specific antibodies against γH2AX (05-636, Millipore), H2AX (ab11175, abcam), phospho-CHK1(S345) (2348, Cell Signaling Technology), CHK1 (sc-8408, Santa Cruz Biotechnology), phospho-CHK2 (T68) (2661, Cell Signaling Technology), CHK2 (sc-5278, Santa Cruz Biotechnology), Caspase 3 (9662, Cell Signaling Technology), and FOXM1 (sc-500, Santa Cruz Biotechnology). All images were acquired by the GeneGnome 5 (Bio Image, Syngene) and the γH2AX/H2AX, p-CHK1/CHK1, and p-CHK2/CHK2 ratios were quantitative by a GeneTools software program (Syngene). These data were repeated at least three times and similar trends were observed.

### Sister chromatid exchange (SCE)

10^6^ cells were incubated with 9μg/mL 5-bromodeoxyuridine (BrdU) (Sigma) for 48 hours, and SCE analysis was performed as described previously. Images were acquired by Nikon eclipse 80i / NIS Elements D4.20.00. For each cell line, 50 metaphases were analyzed to determine the SCE frequency.

### Immunofluorescence microscopy

Cells plated on two-well chamber slides were chronically treated with 5 μM, 10μM, or 20μM cisplatin for 4 or 24 hours and then fixed with 3.5% paraformaldehyde for 15 min and permeabilized with 1% Triton X-100 for 10 min. Fixed cells were blocked with 5% FBS and stained with anti-γH2AX and Alexa-conjugated anti-mouse secondary antibodies. Images were acquired by confocal microscope OLYMPUS IX81. The fluorescence intensity of γH2AX in a cell was quantified by an OLYMPUS FLUOVIEW: FV10-ASW software. At least 100 cells from each cell lines were quantified.

## SUPPLEMENTARY FIGURES


