# Supplementary material for: Chronic treatment with cisplatin induces replication-dependent sister chromatid recombination to confer cisplatin-resistant phenotype in nasopharyngeal carcinoma
Source: Oncotarget. 2014 Jul 12;5(15):6323–37. doi: 10.18632/oncotarget.2210 (PMC4171633; doi:10.18632/oncotarget.2210)
Supplement: Supplementary file 1 [file oncotarget-05-6323-s001.pdf]

Chronic treatment with cisplatin induces replication-dependent sister chromatid recombination to confer cisplatin-resistant phenotype in nasopharyngeal carcinoma

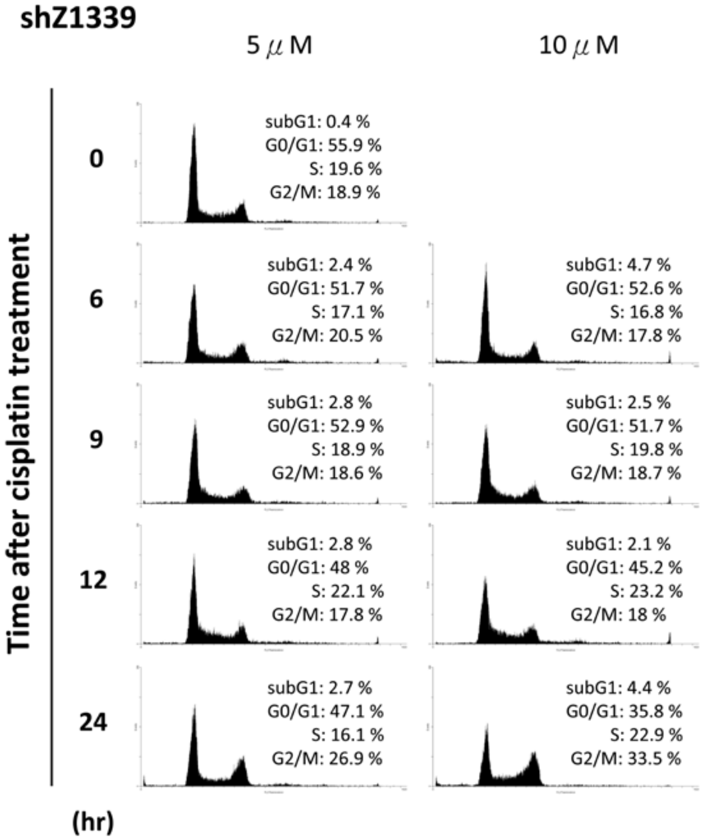

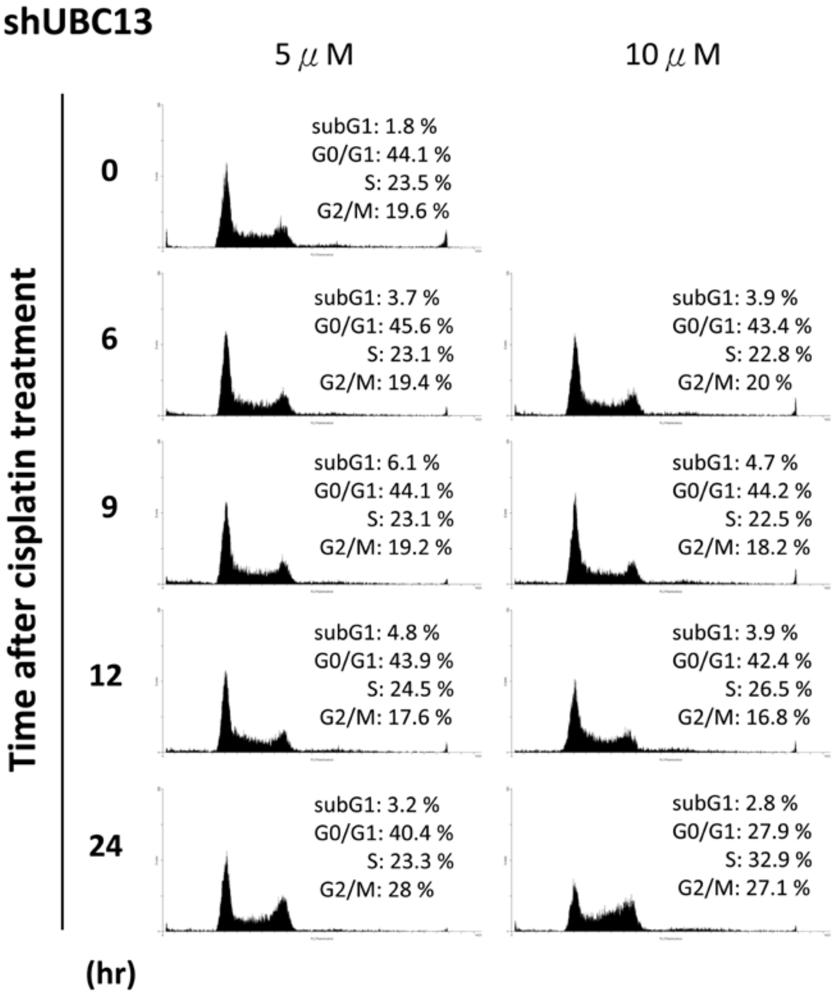

shUBC13
5 μM
10 μM
Time after cisplatin treatment
0
subG1: 1.8 %
G0/G1: 44.1 %
S: 23.5 %
G2/M: 19.6 %
6
subG1: 3.7 %
G0/G1: 45.6 %
S: 23.1 %
G2/M: 19.4 %
subG1: 3.9 %
G0/G1: 43.4 %
S: 22.8 %
G2/M: 20 %
9
subG1: 6.1 %
G0/G1: 44.1 %
S: 23.1 %
G2/M: 19.2 %
subG1: 4.7 %
G0/G1: 44.2 %
S: 22.5 %
G2/M: 18.2 %
12
subG1: 4.8 %
G0/G1: 43.9 %
S: 24.5 %
G2/M: 17.6 %
subG1: 3.9 %
G0/G1: 42.4 %
S: 26.5 %
G2/M: 16.8 %
24
subG1: 3.2 %
G0/G1: 40.4 %
S: 23.3 %
G2/M: 28 %
subG1: 2.8 %
G0/G1: 27.9 %
S: 32.9 %
G2/M: 27.1 %
(hr)

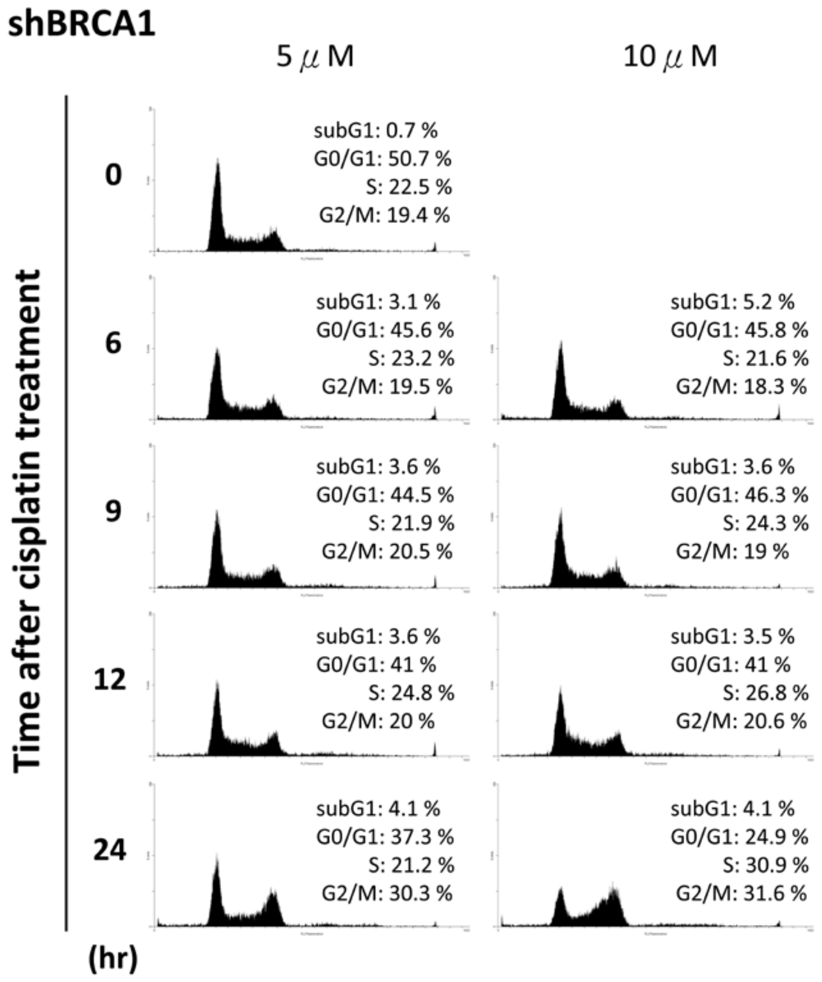
shBRCA1
5 μM
10 μM
Time after cisplatin treatment
0
subG1: 0.7 %
G0/G1: 50.7 %
S: 22.5 %
G2/M: 19.4 %
6
subG1: 3.1 %
G0/G1: 45.6 %
S: 23.2 %
G2/M: 19.5 %
subG1: 5.2 %
G0/G1: 45.8 %
S: 21.6 %
G2/M: 18.3 %
9
subG1: 3.6 %
G0/G1: 44.5 %
S: 21.9 %
G2/M: 20.5 %
subG1: 3.6 %
G0/G1: 46.3 %
S: 24.3 %
G2/M: 19 %
12
subG1: 3.6 %
G0/G1: 41 %
S: 24.8 %
G2/M: 20 %
subG1: 3.5 %
G0/G1: 41 %
S: 26.8 %
G2/M: 20.6 %
24
subG1: 4.1 %
G0/G1: 37.3 %
S: 21.2 %
G2/M: 30.3 %
subG1: 4.1 %
G0/G1: 24.9 %
S: 30.9 %
G2/M: 31.6 %
(hr)

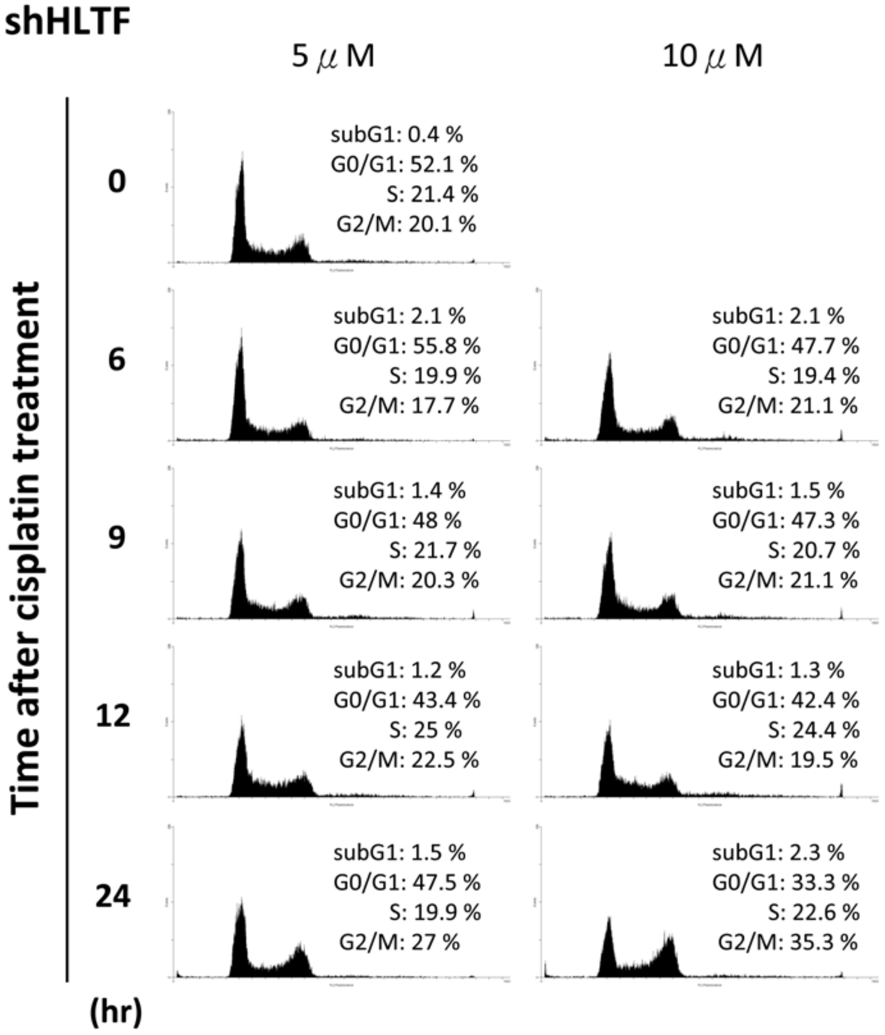
shHLTF
5 μM
10 μM
Time after cisplatin treatment
0
6
9
12
24
(hr)
subG1: 0.4 %
G0/G1: 52.1 %
S: 21.4 %
G2/M: 20.1 %
subG1: 2.1 %
G0/G1: 55.8 %
S: 19.9 %
G2/M: 17.7 %
subG1: 2.1 %
G0/G1: 47.7 %
S: 19.4 %
G2/M: 21.1 %
subG1: 1.4 %
G0/G1: 48 %
S: 21.7 %
G2/M: 20.3 %
subG1: 1.5 %
G0/G1: 47.3 %
S: 20.7 %
G2/M: 21.1 %
subG1: 1.2 %
G0/G1: 43.4 %
S: 25 %
G2/M: 22.5 %
subG1: 1.3 %
G0/G1: 42.4 %
S: 24.4 %
G2/M: 19.5 %
subG1: 1.5 %
G0/G1: 47.5 %
S: 19.9 %
G2/M: 27 %
subG1: 2.3 %
G0/G1: 33.3 %
S: 22.6 %
G2/M: 35.3 %

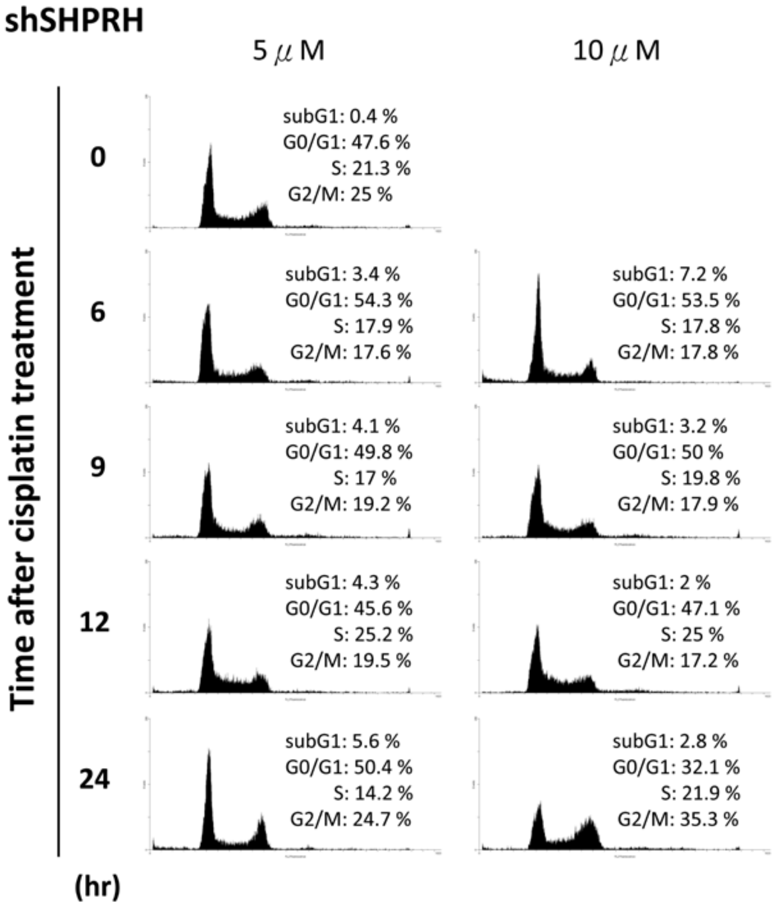
shSHPRH
5 μM
10 μM
Time after cisplatin treatment
0
subG1: 0.4 %
G0/G1: 47.6 %
S: 21.3 %
G2/M: 25 %
6
subG1: 3.4 %
G0/G1: 54.3 %
S: 17.9 %
G2/M: 17.6 %
subG1: 7.2 %
G0/G1: 53.5 %
S: 17.8 %
G2/M: 17.8 %
9
subG1: 4.1 %
G0/G1: 49.8 %
S: 17 %
G2/M: 19.2 %
subG1: 3.2 %
G0/G1: 50 %
S: 19.8 %
G2/M: 17.9 %
12
subG1: 4.3 %
G0/G1: 45.6 %
S: 25.2 %
G2/M: 19.5 %
subG1: 2 %
G0/G1: 47.1 %
S: 25 %
G2/M: 17.2 %
24
subG1: 5.6 %
G0/G1: 50.4 %
S: 14.2 %
G2/M: 24.7 %
subG1: 2.8 %
G0/G1: 32.1 %
S: 21.9 %
G2/M: 35.3 %
(hr)

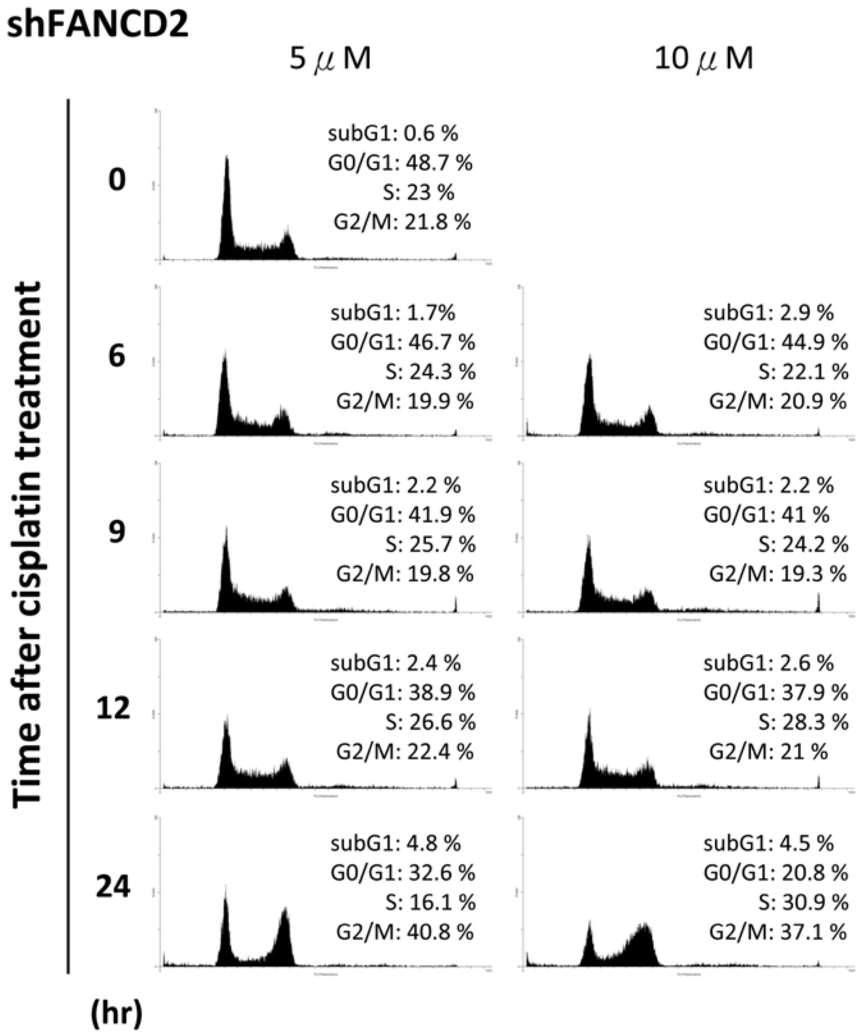

Figure S1: Flow cytometry of control (shZ1339), BRCA1- (shBRCA1), FANCD2- (shFANCD2), HLTF- (shHLTF), SHPRH- (shSHPRH), and UBC13- (shUBC13) deficient cells in the presence of 5 or 10μM cisplatin treatment. Cells were harvested at indicated time points for FACS analysis.

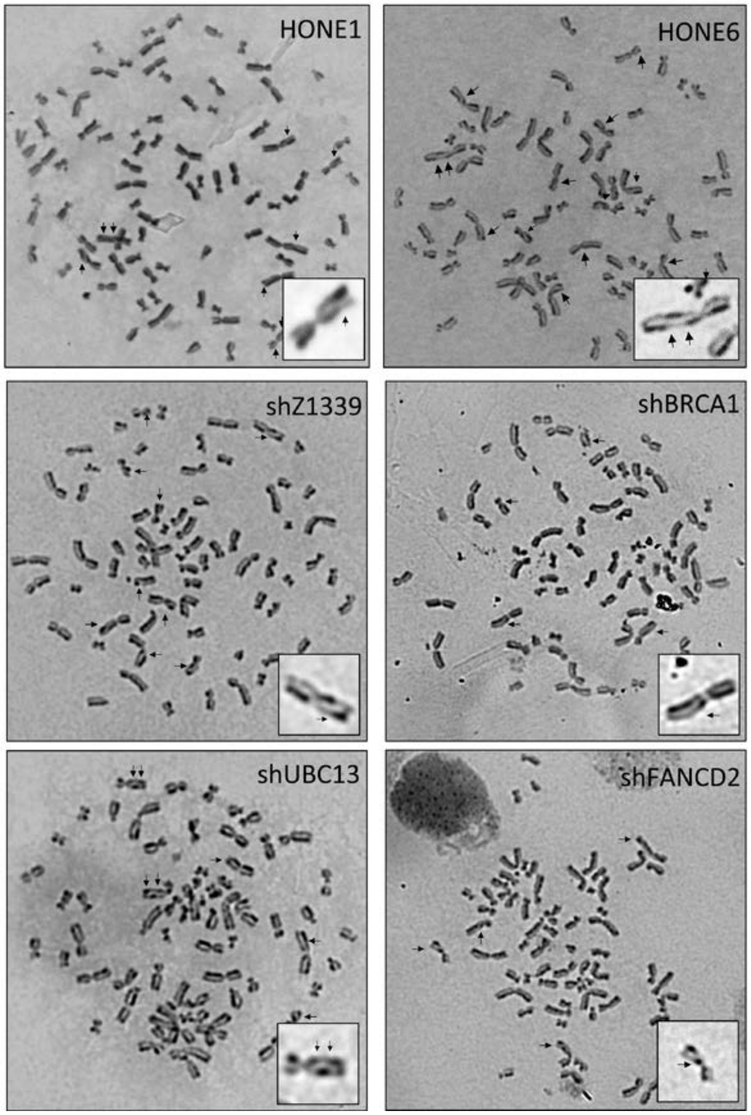

Figure S2: The SCE analysis of HONE1, HONE6, control (shZ1339), BRCA1- (shBRCA1), FANCD2- (shFANCD2), and UBC13- (shUBC13) deficient cells. SCE was scored in 50 metaphases of cells.

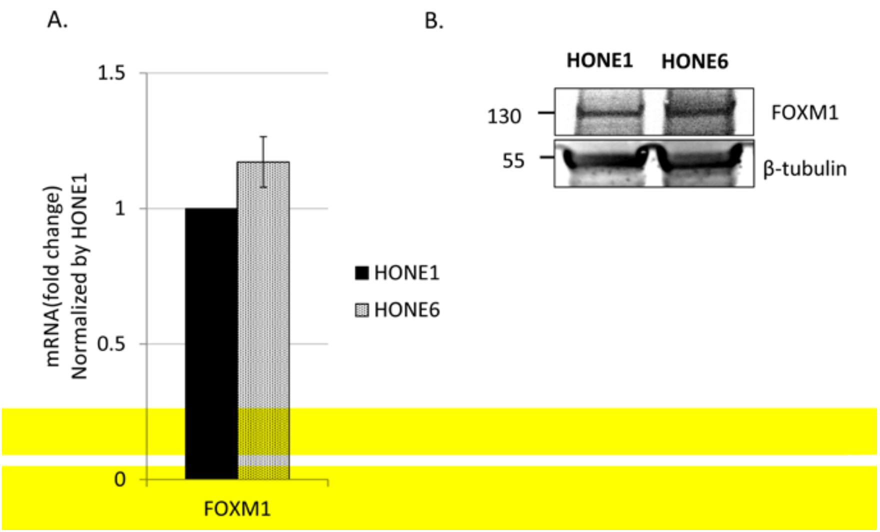

Figure S3: The expression level of FOXM1 is slightly increased in HONE6 cells. (A) The expression level of FOXM1 in HONE1 and HONE6 cells was determined by qRT-PCR and normalized by the level in HONE1 cells. The expression of β-actin (ACTB) was used as an internal control. The expression level of FOXM1 in HONE1 or HONE6 was normalized by the level of ACTB in each cell. (B) The Western blot was immunostained with a specific FOXM1 antibody as indicated.
